# Development and validation of a prognostic model incorporating texture analysis derived from standardised segmentation of PET in patients with oesophageal cancer

**DOI:** 10.1007/s00330-017-4973-y

**Published:** 2017-08-02

**Authors:** Kieran G Foley, Robert K. Hills, Beatrice Berthon, Christopher Marshall, Craig Parkinson, Wyn G. Lewis, Tom D. L. Crosby, Emiliano Spezi, Stuart Ashley Roberts

**Affiliations:** 1 Division of Cancer & Genetics, Cardiff University, Cardiff, UK; 2 Haematology Clinical Trials Unit, Cardiff University, Cardiff, UK; 3 Wales Research & Diagnostic PET Imaging Centre, Cardiff, UK; 4 School of Engineering, Cardiff University, Cardiff, UK; 5 Department of Upper GI Surgery, University Hospital of Wales, Cardiff, UK; 6 Department of Oncology, Velindre Cancer Centre, Cardiff, UK; 7 Department of Clinical Radiology, University Hospital of Wales, Cardiff, UK

**Keywords:** Neoplasms, Oesophagus, Prognosis, Positron-emission tomography, Survival

## Abstract

**Objectives:**

This retrospective cohort study developed a prognostic model incorporating PET texture analysis in patients with oesophageal cancer (OC). Internal validation of the model was performed.

**Methods:**

Consecutive OC patients (*n* = 403) were chronologically separated into development (*n* = 302, September 2010-September 2014, median age = 67.0, males = 227, adenocarcinomas = 237) and validation cohorts (*n* = 101, September 2014-July 2015, median age = 69.0, males = 78, adenocarcinomas = 79). Texture metrics were obtained using a machine-learning algorithm for automatic PET segmentation. A Cox regression model including age, radiological stage, treatment and 16 texture metrics was developed. Patients were stratified into quartiles according to a prognostic score derived from the model. A *p*-value < 0.05 was considered statistically significant. Primary outcome was overall survival (OS).

**Results:**

Six variables were significantly and independently associated with OS: age [HR =1.02 (95% CI 1.01-1.04), *p* < 0.001], radiological stage [1.49 (1.20-1.84), *p* < 0.001], treatment [0.34 (0.24–0.47), *p* < 0.001], log(TLG) [5.74 (1.44–22.83), *p* = 0.013], log(Histogram Energy) [0.27 (0.10–0.74), *p* = 0.011] and Histogram Kurtosis [1.22 (1.04–1.44), *p* = 0.017]. The prognostic score demonstrated significant differences in OS between quartiles in both the development (X^2^ 143.14, df 3, *p* < 0.001) and validation cohorts (X^2^ 20.621, df 3, *p* < 0.001).

**Conclusions:**

This prognostic model can risk stratify patients and demonstrates the additional benefit of PET texture analysis in OC staging.

***Key points*:**

• *PET texture analysis adds prognostic value to oesophageal cancer staging*.

• *Texture metrics are independently and significantly associated with overall survival*.

• *A prognostic model including texture analysis can help risk stratify patients*.

**Electronic supplementary material:**

The online version of this article (doi:10.1007/s00330-017-4973-y) contains supplementary material, which is available to authorized users.

## Introduction

Medical imaging is a fundamental component of cancer staging worldwide and forms a substantial part of current prognostic stratification tools. Innovative radiological techniques are expected to have a substantial role in developing future risk stratification models, which may subsequently influence clinical decision-making.

Cross-sectional imaging allows three-dimensional (3D) tumour visualisation, enabling non-invasive, quantitative analysis of tumour heterogeneity [1]. Texture analysis of medical images, together with other feature extraction algorithms, provides ‘radiomic’ data, which contain first-, second- and higher-order statistics that quantify the spatial distribution and intensity values of voxels within the tumour [2, 3].

Multiple sub-clonal populations of cells are known to co-exist within tumours [4]. Texture analysis could act as a 3D surrogate marker of underlying tumour heterogeneity. This, in combination with traditional staging methods, may improve decision tools and optimise treatment pathways [1].

Retrospective studies have investigated the ability of PET texture analysis to predict treatment response and survival in different solid cancers including lung, oesophageal, cervical, and head and neck [5–7]. A large multi-centre study including 1019 patients with lung and head and neck cancer conducted retrospective radiomic analysis on external data sets and demonstrated the additional benefit of CT texture analysis in the staging pathway. Radiomic data were combined with genomic data to produce a prognostic signature resulting in improved prognostic performance compared to traditional Tumour Node Metastasis (TNM) staging alone [1].

This study aimed to demonstrate the additional prognostic value of PET texture analysis compared with the current staging methods by developing a prognostic model in patients with oesophageal cancer (OC). We aimed to calculate a prognostic score that can stratify patients accordingly and perform internal validation of the prognostic model in an independent cohort of patients.

## Materials and methods

### Patient cohort

This is a retrospective cohort study of consecutive patients with biopsy-proven OC, including gastro-oesophageal junctional (GOJ) tumours, radiologically staged between 16 September 2010 and 31 July 2015. All patients were identified at the Regional Upper Gastro-intestinal (GI) Cancer multi-disciplinary team (MDT) meeting. Institutional Review Board approval was granted and the requirement for informed consent was waived.

Overall, 550 patients were considered for inclusion. Exclusion criteria were non- or poorly FDG-avid tumours [SUV_max_ <3 (*n* = 60)], an MTV <5 ml (*n* = 52), histology other than adenocarcinoma or squamous cell carcinoma (*n* = 21), a synchronous primary malignancy (*n* = 7) or an oesophageal stent in situ (*n* = 7).

Following exclusions, 403 patients were included and chronologically separated into two independent cohorts. The first (development) cohort included 302 patients radiologically staged between 16 September 2010 and 15 September 2014. The second (validation) cohort included 101 patients radiologically staged between 16 September 2014 and 31 July 2015.

All patients were deemed to have potentially curable disease following contrast-enhanced CT (CECT) staging investigation. All PET/CT examinations were performed separately, following the initial CECT, and reported in the same centre by Consultant Radiologists with an interest in Nuclear Medicine. Clinical, radiological, histological and outcome data were recorded in a prospectively maintained database and were updated in July 2016. Radiological staging was performed according to the Union for International Cancer Control (UICC) TNM 7th edition [8].

### PET/CT protocol

Patients were fasted for at least 6 hours prior to tracer administration. Serum glucose levels were routinely checked and confirmed as less than 7.0 mmol/L prior to imaging. Patients received a dose of 4 MBq of ^18^F-FDG/kg. Uptake time was 90 min, standard practice at our institution. A GE 690 scanner (GE Healthcare, Buckinghamshire, UK) was used. CT images were acquired in a helical acquisition with a pitch of 0.98 and tube rotation speed of 0.5 s. Tube output was 120 kVp with output modulation between 20 and 200 mA. Matrix size for the CT acquisition was 512 × 512 pixels with a 50-cm field of view. No oral or intravenous contrast was administered. PET images were acquired at 3 min per field of view. The length of the axial field of view was 15.7 cm (skull base to mid-thigh). Images were reconstructed with the ordered subset expectation maximisation algorithm, with 24 subsets and 2 iterations. Matrix size was 256 × 256 pixels, using the VUE Point™ time of flight algorithm.

### Treatment protocols

Patients had surgery alone (SA), neo-adjuvant chemotherapy (NACT) or neo-adjuvant chemoradiotherapy (NACRT) prior to surgery, definitive chemo-radiotherapy (dCRT) or palliative therapy. The optimum treatment strategy was decided by consensus at the MDT. In general, fit patients with tumours pre-operatively staged as T3/T4a, N0/N1 were pre-operatively treated with NACT or NACRT. Less fit patients, or those with T1/2 N0 disease, had surgery alone. Patients deemed unsuitable for surgery because of co-morbidity and/or performance status, extensive loco-regional disease or personal choice received dCRT.

### Data preparation and PET segmentation

**Fig. 1:**
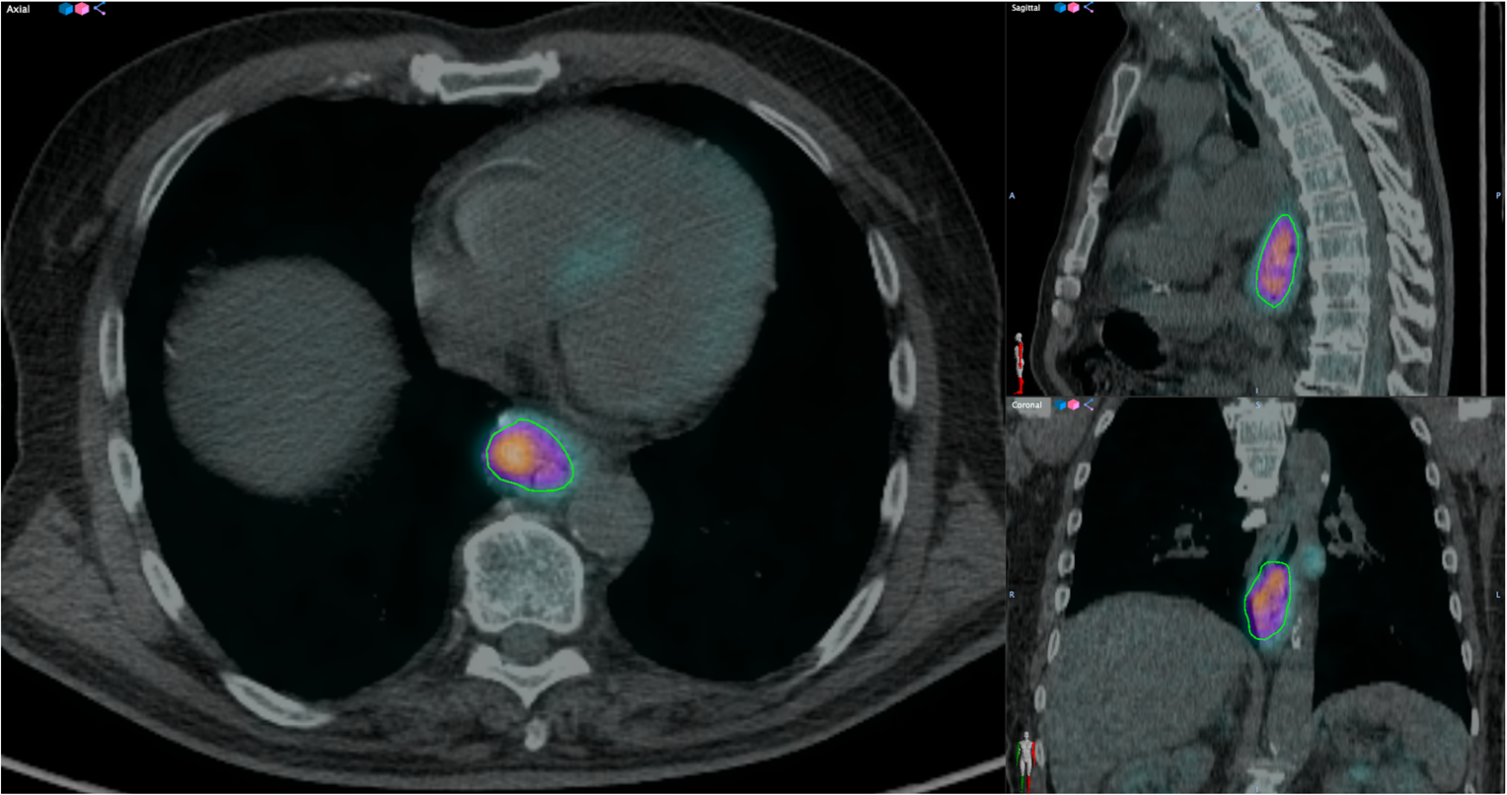
Fused axial, sagittal and coronal FDG-PET/CT images of an oesophageal tumour outlined with ATLAAS segmentation tool

Texture analysis of PET images is dependent on the segmentation method used to define the metabolic tumour volume (MTV) [3]. A novel tool called ATLAAS (Automatic Decision Tree Learning Algorithm for Advanced Segmentation) has been developed to standardise segmentation of PET images [9]. Data preparation was performed by a radiology resident (KF) with 4 years’ experience of PET research who was blinded to clinical data. ATLAAS segmentation was applied using a graphical user interface (GUI) written in the MatLab language as a plug-in to the Computational Environment for Radiotherapy Research (CERR) [10] (Fig. 1). ATLAAS segmentation first requires creation of a bounding box, which was manually performed in each case. The time taken to perform this process varies, depending on the MTV and proximity to other FDG-avid organs, but can take just a few minutes. Adjustment of the window level and colour of displayed PET images was performed at the discretion of the user, but no pre-defined levels were used since these have no influence on ATLAAS segmentation. The adequacy of ATLAAS segmentation was confirmed by visual assessment in each case. PET images were re-sampled into 0.5 SUV bins. This method is recommended because SUVs are distributed into equally sized intensity bins [11].

### Prognostic variables

Nineteen variables were included in the Cox regression model. Age (number of years) and stage group (I A or B = 1, II A or B = 2, III A, B or C = 3, IV = 4) were included. Treatment was divided into curative (=1) and palliative (=2) groups prior to data analysis. SUV_max_ and SUV_mean_ are the maximum and mean SUV, respectively [12]. MTV derived from ATLAAS and tumour lesion glycolysis (TLG), the product of SUV_mean_ and MTV, were calculated [12]. First-order histogram metrics including Histogram Standard Deviation, Histogram Entropy, Histogram Energy, Histogram Skewness and Histogram Kurtosis, were implemented as in Orlhac et al. [5]. Grey-level co-occurrence matrix-based (GLCMs) metrics including Homogeneity, Entropy and Dissimilarity were implemented as in Haralick et al. [13]. Coarseness, implemented as in Amadasun et al. [14], was calculated, along with grey-level size zone matrices (GLSZMs), Intensity Variability, Large Area Emphasis and Zone Percentage, which were implemented as in Thibault et al. [15]. These texture metrics have been selected for inclusion in this study as they have shown prognostic and predictive significance in other texture analysis studies investigating OC [16–18].

### Transformation of variables

Visual inspection of continuous variable histograms was performed before model development to assess for normal distribution and skewness. Specific normality tests were not used but logarithmic transformation of variables with significant long tails was performed prior to analysis to reduce the leverage created from outlying data. Four texture variables were transformed: TLG [log(TLG)], Histogram Energy [log(Energy)], Coarseness [log(Coarseness)] and Homogeneity [log(Homogeneity)]. Repeat inspection of the transformed histograms revealed the four variables had a more normalised distribution.

### Metabolic tumour volume and texture metrics

An important consideration in texture analysis is the range of tumour volumes assessed. Tumours with small volumes may provide redundant texture information because of highly correlated variables [19]. Some authors have suggested excluding tumours with MTV less than 5 ml [5]. Therefore, patients with MTVs <5 ml were excluded from the analysis.

### Outcome data

The primary outcome of the study is OS, defined as number of months survived from date of diagnosis. Patients are followed up 3 monthly for the 1st year, 6 monthly until 5 years then annually thereafter, or until death. All included patients were followed-up for at least 12 months. Date of death was obtained from the Cancer Network Information System Cymru database (CaNISC, Velindre NHS Trust, Wales).

### Statistical analysis

Categorical variables were described as frequency (percent) and continuous variables as median (range) and differences assessed with appropriate non-parametric tests. Cumulative survival was calculated by the Kaplan-Meier life-table method. A Cox Regression Model with a backward conditional method was constructed by an experienced medical statistician. Model power was based on an event-to-variable ratio (EPV), recommended to be a minimum level of 10 [20]. EPV is defined as the ratio of the number of patient deaths compared to the number of variables in the model. The prognostic score was calculated by summation of the products of variables and their corresponding parameter estimate. Using this, patients were separated into quartiles and a log-rank test evaluated significant differences in OS. The effect of curative or palliative treatment on the performance of the prognostic score was assessed with a test of interaction. Furthermore, the Akaike information criterion (AIC) statistic evaluated the estimated quality of three incremental models: (1) a model including age, radiological stage group and treatment; (2) a model including these variables plus newer prognostic indicators SUV_max_, SUV_mean_ and MTV; (3) a model including the additional texture metrics. AIC is calculated by −2*log(L) + 2k, where k is the number of parameters and L is the likelihood of the model [21]. The model with the lowest AIC value is considered the better model. Internal validation of the prognostic model was performed retrospectively in a separate cohort of patients. A *p*-value of < 0.05 was considered statistically significant. Statistical analysis was performed using SAS version 9.4 (SAS, Cary, NC, USA) and SPSS version 23.0 (IBM, Chicago, IL, USA).

## Results

Baseline characteristics of patients included in the development and validation cohorts are detailed in Table 1. The median OS of the development and validation cohorts was 16.0 months [95% confidence interval (95% CI) 13.8-18.2] and 14.0 months (95% CI 10.4-17.6), respectively. Median follow-up was 43.0 months (95% CI 35.3-50.7) in the development cohort and 17.0 months (95% CI 15.7-18.3) in the validation cohort. Overall 1- and 2-year survival in the development cohort was 66.9% and 33.3%, respectively, and 1-year OS in the validation cohort was 57.4%. Classification of the radiological EUS and PET/CT TNM stage is detailed in electronic supplementary material, S1.

**Table 1:** Baseline characteristics of patients in development and validation cohorts

Frequency (%)	Development cohort (*n* = 302)	Validation cohort (*n* = 101)	*p*-value*
Median age	67.0 years (range 39–83)	69.0 years (range 39–84)	0.179
Gender (M:F)	227 (75.2): 75 (24.8)	78 (77.2): 23 (22.8)	0.676
Histology			0.956
Adenocarcinoma	237 (78.5)	79 (78.2)	
Squamous cell Carcinoma	65 (21.5)	22 (21.8)	
Tumour location			0.003
Oesophagus	192 (63.6)	47 (46.5)	
Upper third	6 (3.1)	3 (6.4)	
Middle third	53 (27.6)	10 (21.3)	
Lower third	133 (69.3)	34 (72.3)	
Junction	110 (36.4)	54 (53.5)	
Siewert I	41 (37.3)	24 (44.5)	
Siewert II	30 (27.3)	18 (33.3)	
Siewert III	39 (35.4)	12 (22.2)	
Stage groups			0.238
Stage 1	17 (5.6)	2 (2.0)	
Stage 2	56 (18.5)	24 (23.8)	
Stage 3	160 (53.1)	57 (56.4)	
Stage 4	69 (22.8)	18 (17.8)	
Treatment			0.624
Curative	158 (52.3)	50 (49.5)	
SA	24 (15.2)	4 (8.0)	
NACT	67 (42.4)	23 (46.0)	
NACRT	13 (8.2)	7 (14.0)	
dCRT	54 (34.2)	16 (32.0)	
Palliative	144 (47.7)	51 (50.5)	
Overall survival			<0.001
Alive	70 (23.2)	43 (42.6)	
Dead	232 (76.8)	58 (57.4)	

*SA* surgery alone; *NACT* neo-adjuvant chemotherapy; *NACRT* neo-adjuvant chemoradiotherapy; *dCRT* definitive chemo-radiotherapy; *chi-square or Mann–Whitney U test

### Prognostic model development

The final step of the prognostic model is presented in Table 2. Descriptive statistics for all calculated PET metrics are detailed in electronic supplementary material, S2. There were 232 events and 19 variables in the model, providing 12.2 EPV. In addition to known important prognostic factors in OC (age, radiological stage and treatment), the model identified 3 texture metrics that were independently and significantly associated with survival. The significant variables were log(TLG), log(Histogram Energy) and Histogram Kurtosis. Their inclusion in the model illustrates their additional prognostic value compared with current prognostic factors. TLG is calculated as the product of SUV_mean_ and MTV [12]. Histogram Energy [5] was calculated using Eq. 1:

**Table 2:** Results of the Cox regression model

Prognostic variable	*p*-value	Parameter estimate	Hazard ratio	95% Confidence limits	
				Lower	Upper
TNM stage	<0.001	0.397	1.49	1.20	1.84
Treatment	<0.001	−1.094	0.34	0.24	0.47
Age	0.001	0.024	1.02	1.01	1.04
log(Histogram Energy)	0.011	−1.320	0.27	0.10	0.74
log(TLG)	0.013	1.748	5.74	1.44	22.83
Histogram Kurtosis	0.017	0.198	1.22	1.04	1.44

1 $$ Histogram\kern0.5em Energy={\varSigma}_i{\left(P(i)\right)}^2 $$

where $P(i)=\frac{N_i}{N},$ with ${N}_i$ the number of voxels of intensity I and N the total number of voxels. Histogram Kurtosis [5] was calculated using Eq. 2:

2 $$ Histrogram\kern0.5em  Kurtosis=\frac{\frac{1}{N}{\varSigma}_i{\left(I(i)-\mu \right)}^4}{{\left(\frac{1}{N}{\varSigma}_i{\left(I(i)-\mu \right)}^2\right)}^2} $$

where N is the number of voxels in the image, I(i) is the positive intensity value in the 3D matrix, and μ is the mean intensity value.

### Prognostic score calculation

**Fig. 2:**
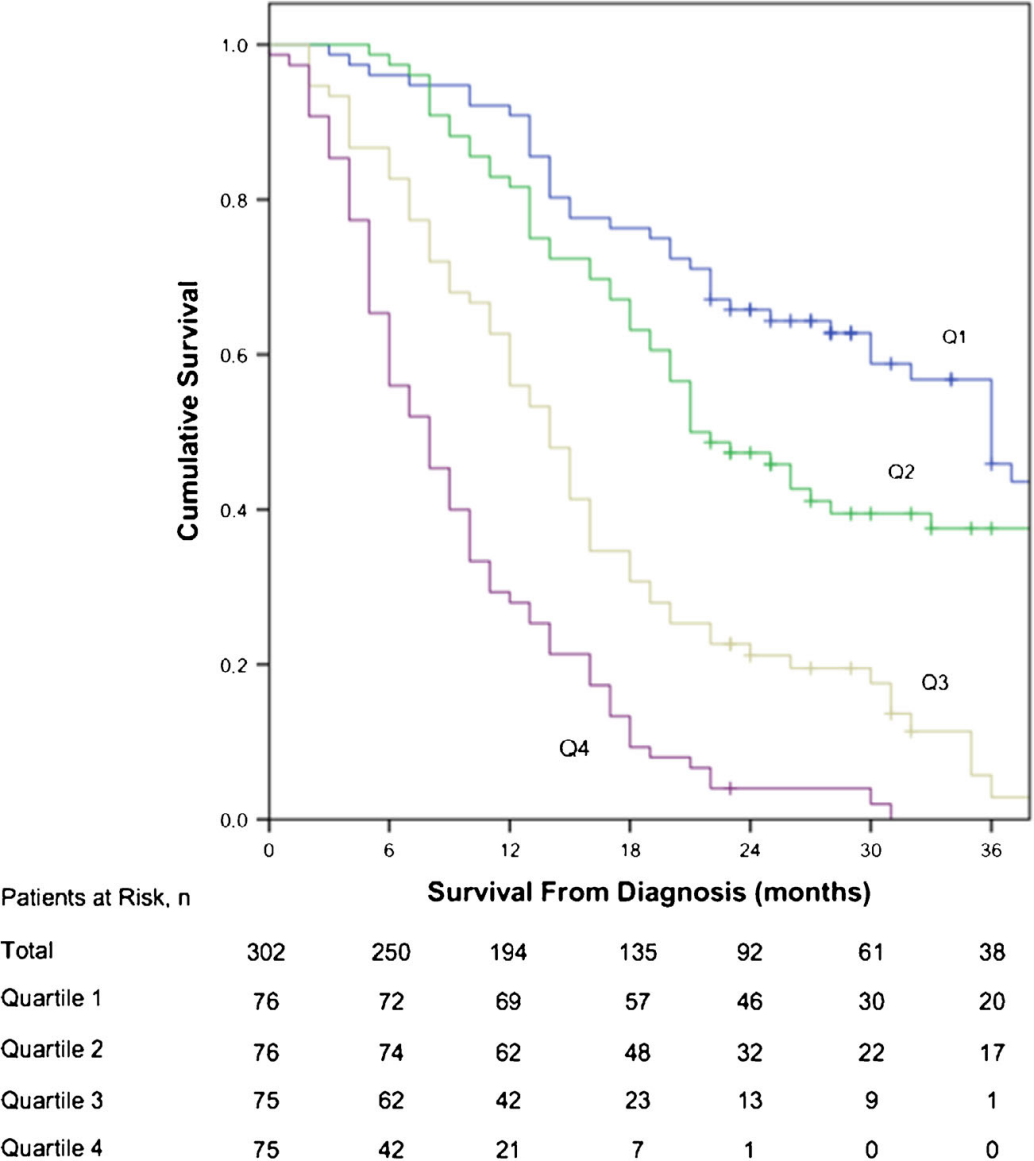
Kaplan-Meier plot demonstrating overall survival curves of prognostic score quartiles in the development group (X^2^ 143.14, df 3, *p* < 0.001). Q1 quartile; Q2 quartile 2; Q3 quartile 3; Q4 quartile 4. Median OS of Q1 to Q4 was 36.0 months (95% CI 31.1-40.9), 21.0 months (16.1-25.9), 14.0 months (11.7-16.3) and 8.0 months (5.9-10.1), respectively

The equation used to calculate the prognostic score in the development cohort was (Stage Group*0.397) - (Treatment*1.094) + (Age*0.024) - (log(Histogram Energy)*1.320) + (log(TLG)*1.748) + (Histogram Kurtosis*0.198). This calculation was derived using published methods [22]. The median score of quartile 1 was −0.73 (*n* = 76, range −1.66 to −0.45), quartile 2 was −0.14 (*n* = 76, −0.45 to 0.29), quartile 3 was 0.76 (*n* = 75, 0.31 to 1.06) and quartile 4 was 1.38 (*n* = 75, 1.08 to 2.15). There was a significant difference in OS between quartiles (X^2^ 143.14, df 3, *p* < 0.001) (Fig. 2). Median OS of quartiles 1 to 4 was 36.0 months (95% CI 31.1-40.9), 21.0 months (16.1-25.9), 14.0 months (11.7-16.3) and 8.0 months (5.9-10.1), respectively. The interaction test revealed no statistical difference in performance of the prognostic score between curative and palliative treatments (X^2^ 1.344, df 1, *p* = 0.246).

### Comparison of estimated model performance

The AIC of the traditional model including the radiological stage group, treatment and age was 2247.693. The AIC of the model that also included SUV_max_, SUV_mean_ and MTV was also 2247.693. The AIC of the development prognostic model including additional texture metrics was 2238.007, which was the lowest value. This suggests that incorporation of PET variables and texture metrics improves current prognostic models in OC.

### Internal validation of prognostic model

**Fig. 3:**
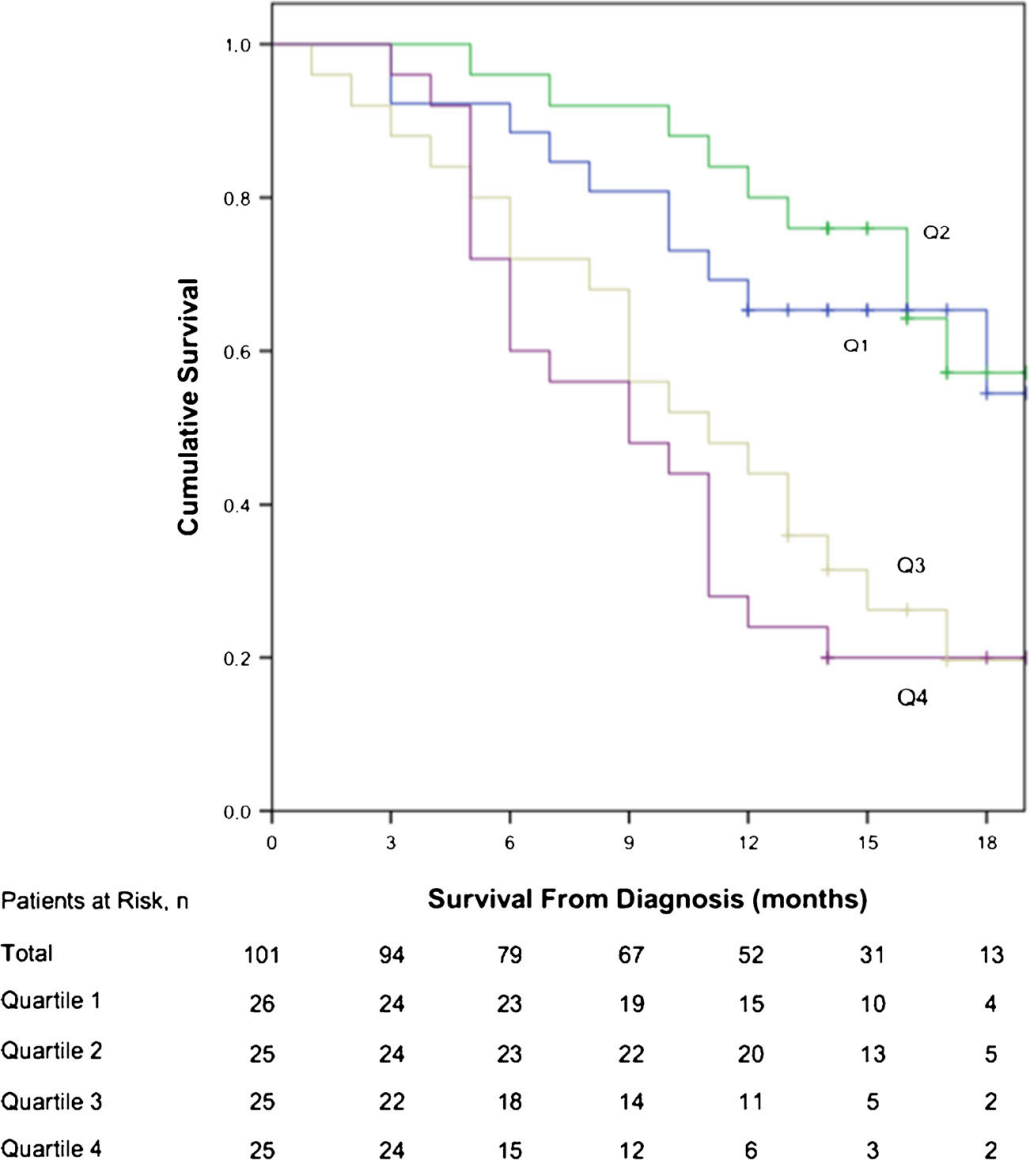
Kaplan-Meier plot demonstrating overall survival curves of prognostic score quartiles in the validation group (X^2^ 20.621, df 3, *p* < 0.001). Q1 quartile; Q2 quartile 2; Q3 quartile 3; Q4 quartile 4. Mean OS of patients in Q1 and Q2 was 16.6 months (95% CI 13.9-19.3) and 17.4 months (15.4-19.4), respectively. Median OS for Q3 and Q4 was 11.0 months (6.1-15.9) and 9.0 months (4.1-13.9), respectively

The prognostic model was applied to the validation cohort. Again, there was a significant difference in OS between patient quartiles (X^2^ 20.621, df 3, *p* < 0.001) (Fig. 3). Results of PET metrics obtained from the validation cohort are detailed in electronic supplementary material, S3. Mean OS of patients in quartiles 1 and 2 was 16.6 months (95% CI 13.9-19.3) and 17.4 months (15.4-19.4), respectively. Patients in quartile 1 had lower mean OS than those in quartile 2, but the difference between quartiles was not significant (X^2^ = 0.219, df =1, *p* = 0.640). The median OS for quartiles 3 and 4 was 11.0 months (6.1-15.9) and 9.0 months (4.1-13.9). Three of 26 (11.5%) patients were treated with palliative intent in quartile 2, and 2 of 25 (8.0%) patients were treated with curative intent in quartile 3. The AIC of the validation model including PET variables and texture metrics was lower (464.671) than in models including the radiological stage group, treatment and age (470.420), and SUV_max_, SUV_mean_ and MTV (470.420), respectively.

## Discussion

This study has developed a prognostic model that provides new and important results for OC staging. Internal validation of the model demonstrated a continued difference in OS (*p* < 0.001) between quartiles in an independent cohort of patients. The results of this study show that PET texture analysis may enhance the prognostic TNM staging model in OC.

The prognostic model has identified three PET metrics: log(TLG), log(Histogram Energy) and Histogram Kurtosis, which are significantly and independently associated with OS. These metrics have added value over and above currently known prognostic factors: age, radiological stage and treatment. These findings indicate the additional value of novel texture analysis methods in modern staging pathways, which was confirmed with the AIC statistic. Improved risk-stratification could identify sub-groups of patients in which a certain treatment may improve OS [23] or where a therapeutic intervention may be ineffective or harmful [24].

According to the model, patients with increased log(TLG) and Histogram Kurtosis, and reduced log(Histogram Energy), have an increased likelihood of mortality. Raised TLG represents larger, more FDG-avid tumours. The correlation of Histogram Kurtosis and log(Histogram Energy) suggests that tumours with less intensity variation have a worse prognosis. This is an unexpected finding, since it is thought that tumours with more intensity variation result in poorer outcome. Further studies correlating texture features with underlying tumour biology are required to fully understand the interpretation of these metrics [25].

The AIC was identical for traditional TNM and models including SUV and MTV in both the development and validation cohorts. This suggests that SUV and MTV have no additional prognostic value over current staging methods. However, this study has not been designed to test this hypothesis and cannot draw this conclusion.

Our findings concur with other studies in which texture metrics derived from histograms demonstrated significant associations with OS, stage of disease and likelihood of treatment response in OC [16, 26, 27]. However, such studies included fewer patients and used different texture analysis software packages.

Texture metrics are dependent on several parameters [28]. The technical implementation of each metric, the segmentation method used, scan acquisition, image smoothing, influence of quantisation and reconstruction parameters all influence the texture analysis results [3, 11, 29]. There are also limitations specific to PET images, given the relatively large voxel volume and presence of noise artefact [30]. Standardisation of texture analysis techniques are essential for multi-centre comparison studies and development of externally validated prognostic models [11, 31].

In this study, the texture metrics were derived using the ATLAAS algorithm and a standardised workflow was implemented to ensure reproducible and consistent methods. The benefit of ATLAAS is that the best fitting PET automatic segmentation (PET-AS) method is selected in each individual case from a range of segmentation methods that are built into the ATLAAS algorithm. Commonly used PET-AS methods built into the ATLAAS algorithm include adaptive thresholding, Fuzzy C-means (FCM) and region-growing (RG) methods [9]. ATLAAS was originally designed and tested on patients with FDG-avid head and neck tumours. However, it is also applicable to other FDG-avid tumour sites and validation studies are on-going at our institution. Although a new version of ATLAAS had not specifically been designed for this prognostic OC model, visual inspection of the segmented tumour was performed in each case to ensure an appropriate contour had been produced. A benefit of ATLAAS is that tumour segmentation occurs within seconds once the bounding box has been created.

### Strengths of study

This study provides development and internal validation of a prognostic model incorporating PET texture metrics in 403 patients with OC. ATLAAS is a novel machine-learning method that provides robust segmentation results and removes variability by standardising image segmentation. Appropriate statistical methods have been used in this study [23]. The regional upper GI cancer MDT covers a large population of approximately 1.4 million and benefits from the input of highly experienced radiologists, oncologists and surgeons [32].

### Limitations of study

As this study is retrospective, treatment was included in the model and simplified into two groups, curative and palliative. However, the test for interaction showed that the prognostic score could be used in both curative and palliative cohorts with no significant difference in performance. This prognostic model excludes patients with an MTV of less than 5 ml because the quality of the additional data obtained from these models in uncertain [19]. This criterion excludes 11.6% of potential patients from this study. Another prognostic model including small tumour volumes should be developed for these patients but this model is applicable to many patients with FDG-avid oesophageal tumours.

In conclusion, this large study has developed and validated a prognostic model that demonstrates the additional value of PET texture analysis in OC staging. Three PET metrics, log(TLG), log(Histogram Energy) and Histogram Kurtosis, were identified as potentially important variables. These metrics were derived using ATLAAS, a novel machine-learning method designed to optimise and standardise image segmentation. This prognostic model requires further internal and external validation but may be used as a ‘benchmark’ for further studies investigating the value of PET texture analysis in OC. This study highlights the additional benefit of quantitative imaging techniques in cancer staging, which have the potential to improve patient risk stratification.

## Electronic supplementary material

Below is the link to the electronic supplementary material.

ESM 1 (DOCX 85 kb)
