# Supplementary material for: Development and validation of a prognostic model incorporating texture analysis derived from standardised segmentation of PET in patients with oesophageal cancer
Source: Eur Radiol. 2017 Aug 2;28(1):428–36. doi: 10.1007/s00330-017-4973-y (PMC5717119; doi:10.1007/s00330-017-4973-y)
Supplement: Supplementary file 1 — (DOCX 85 kb) [file 330_2017_4973_MOESM1_ESM.docx]

**Electronic Supplementary Material (ESM)**

All EUS examinations were performed in 3 centres by 4 experienced endosonographers. Patients with tumours too stenotic to be crossed at EUS were unable to be fully staged, therefore final pre-treatment radiological stage relied on a combination of findings from PET/CT and CECT investigations. EUS was not attempted if the decision to treat the patient palliatively was made after PET/CT.

In the development cohort, EUS T-stage was assigned in 227 patients (75.2%). EUS N-stage was assigned in 221 patients (73.2%). EUS was not attempted due to M1 disease on PET/CT, or was incomplete due to a non-traversable tumour in 81 cases. In the validation cohort, EUS staging was completed in 78 patients (77.2%). EUS was not attempted due to M1 disease on PET/CT, or was incomplete due to a non-traversable tumour in 23 cases.

S1 TNM Classification of PET/CT and EUS Staging Investigations in Development and Validation Cohorts

| **Frequency (%)** | **Development Cohort (n=302)** | **Validation Cohort (n=101)** | **p-value*** |
| --- | --- | --- | --- |
| EUS T-stage  T1  T2  T3  T4a  T4b  TX | 3 (1.3)  15 (6.6)  161 (71.0)  35 (15.4)  12 (5.3)  1 (0.4) | 1 (1.3)  4 (5.1)  57 (73.1)  15 (19.2)  1 (1.3)  0 (0.0) | 0.656 |
| Total | 227 (100.0) | 78 (100.0) |  |
| EUS N-stage  N0  N1  N2  N3 | 87 (39.4)  59 (26.7)  47 (21.2)  28 (12.7) | 28 (35.9)  36 (46.2)  12 (15.4)  2 (2.5) | 0.003 |
| Total | 221 (100.0) | 78 (100.0) |  |
| PET/CT N-stage  N0  N1  N2  N3 | 126 (41.7)  96 (31.8)  62 (20.5)  18 (6.0) | 46 (45.5)  25 (24.8)  25 (24.8)  5 (4.9) | 0.519 |
| Total | 302 (100.0) | 101 (100.0) |  |
| PET M-stage  M0  M1  MX | 228 (75.5)  72 (23.8)  2 (0.7) | 81 (80.2)  19 (18.8)  1 (1.0) | 0.556 |
| Total | 302 (100.0) | 101 (100.0) |  |

*Chi-square test

S2 Results of PET Variables and Texture Metrics in Development Cohort

| Metric | Mean | 95% Confidence Intervals | | Minimum Value | Maximum Value |
| --- | --- | --- | --- | --- | --- |
|  |  | Lower | Upper |  |  |
| SUV_max_ | 16.55 | 15.57 | 17.54 | 3.56 | 59.97 |
| SUV_mean_ | 9.14 | 8.58 | 9.71 | 2.07 | 35.06 |
| MTV | 25.80 | 23.33 | 28.27 | 5.04 | 132.47 |
| log(TLG) | 2.20 | 2.15 | 2.24 | 1.30 | 3.30 |
| Standard Deviation | 2.51 | 2.35 | 2.66 | 0.46 | 9.73 |
| Histogram Entropy | 3.82 | 3.81 | 3.84 | 2.94 | 4.09 |
| log(Histogram Energy) | 4.74 | 4.68 | 4.81 | 3.52 | 6.37 |
| Histogram Skewness | 0.58 | 0.53 | 0.62 | -0.31 | 2.82 |
| Histogram Kurtosis | 2.81 | 2.70 | 2.92 | 1.72 | 12.69 |
| log(Coarseness) | -1.96 | -1.99 | -1.93 | -2.78 | -1.29 |
| log(Homogeneity) | -0.53 | -0.55 | -0.52 | -1.03 | -0.21 |
| Entropy | 5.34 | 5.25 | 5.43 | 3.10 | 7.25 |
| Dissimilarity | 5.05 | 4.73 | 5.38 | 0.97 | 23.18 |
| Intensity Variability | 18.04 | 16.59 | 19.48 | 2.76 | 90.81 |
| Large Area Emphasis | 258.77 | 78.03 | 439.51 | 1.46 | 20512.36 |
| Zone Percentage | 42.46 | 40.43 | 44.50 | 1.73 | 87.86 |

S3 Results of PET Variables and Texture Metrics in Validation Cohort

| Metric | Mean | 95% Confidence Intervals | | Minimum Value | Maximum Value |
| --- | --- | --- | --- | --- | --- |
|  |  | Lower | Upper |  |  |
| SUV_max_ | 17.13 | 15.02 | 19.23 | 4.57 | 70.97 |
| SUV_mean_ | 9.69 | 8.45 | 10.93 | 2.97 | 39.59 |
| MTV | 25.35 | 21.20 | 29.50 | 5.13 | 100.27 |
| log(TLG) | 2.20 | 2.12 | 2.29 | 1.31 | 3.44 |
| Standard Deviation | 2.54 | 2.21 | 2.87 | 0.27 | 11.11 |
| Histogram Entropy | 3.83 | 3.80 | 3.85 | 3.27 | 4.05 |
| log(Histogram Energy) | 4.76 | 4.64 | 4.88 | 3.45 | 6.49 |
| Histogram Skewness | 0.63 | 0.56 | 0.70 | -0.03 | 1.90 |
| Histogram Kurtosis | 2.79 | 2.65 | 2.93 | 1.85 | 6.14 |
| log(Coarseness) | -1.98 | -2.04 | -1.93 | -2.66 | -1.52 |
| log(Homogeneity) | -0.54 | -0.57 | -0.52 | -1.04 | -0.27 |
| Entropy | 5.37 | 5.21 | 5.52 | 3.45 | 7.37 |
| Dissimilarity | 5.44 | 4.69 | 6.18 | 1.62 | 26.47 |
| Intensity Variability | 18.00 | 15.40 | 20.61 | 4.43 | 75.37 |
| Large Area Emphasis | 107.65 | 38.78 | 176.51 | 1.47 | 2467.89 |
| Zone Percentage | 43.21 | 39.51 | 46.91 | 6.55 | 88.13 |
